# A topically-sprayable, activatable fluorescent and retaining probe, SPiDER-βGal for detecting cancer: Advantages of anchoring to cellular proteins after activation

**DOI:** 10.18632/oncotarget.17080

**Published:** 2017-04-13

**Authors:** Yuko Nakamura, Ai Mochida, Tadanobu Nagaya, Shuhei Okuyama, Fusa Ogata, Peter L. Choyke, Hisataka Kobayashi

**Affiliations:** ^1^ Molecular Imaging Program, Center for Cancer Research, National Cancer Institute, United States National Institutes of Health, Bethesda, Maryland 20892-1088, USA

**Keywords:** activatable probe, β-galactosidase, ovarian cancer, preservation of fluorescence, target-to-background ratios

## Abstract

SPiDER-βGal is a newly-developed probe that is activated by β-galactosidase and is then retained within cells by anchoring to intracellular proteins. Previous work has focused on gGlu-HMRG, a probe activated by *γ*-glutamyltranspeptidase, which demonstrated high sensitivity for the detection of peritoneal ovarian cancer metastases in an animal model. However, its fluorescence, after activation by *γ*-glutamyltranspeptidase, rapidly declines over time, limiting the actual imaging window and the ability to define the border of lesions. The purpose of this study is to compare the fluorescence signal kinetics of SPiDER-βGal with that of gGlu-HMRG using ovarian cancer cell lines *in vitro* and *ex vivo* tissue imaging. *In vitro* removal of gGlu-HMRG resulted in a rapid decrease of fluorescence intensity followed by a more gradual decrease up to 60 min while there was a gradual increase in fluorescence up to 60 min after removal of SPiDER-βGal. This is most likely due to internalization and retention of the dye within cells. This was also confirmed *ex vivo* tissue imaging using a red fluorescence protein (RFP)-labeled tumor model in which the intensity of fluorescence increased gradually after activation of SPiDER-βGal. Additionally, SPiDER-βGal resulted in intense enhancement within the tumor due to the high target-to-background ratio, which extended up to 60 min after activation. In contrast, gGlu-HMRG fluorescence resulted in decreasing fluorescence over time in extracted tumors. Thus, SPiDER-βGal has the advantages of higher signal with more signal retention, resulting in improved contrast of the tumor margin and suggesting it may be an alternative to existing activatable probes.

## INTRODUCTION

The success of oncologic procedures, such as surgery and endoscopy, depends on the rapid and accurate localization of cancers, followed by their complete resection or ablation. Although large tumors are visible to the unaided human eye, tiny foci (< 2 to 3 mm) of cancer may be impossible to see and thus incomplete resections occur increasing the likelihood of recurrence. Consequently, optical fluorescence-guided imaging is being investigated as a tool to assist physicians during oncologic resections.

Activatable fluorescent probes are designed to become fluorescent only after they come in contact with the target tissue and are activated by particular conditions such as enzymes, pH, temperature etc. This type of optical probe inherently results in lower background signals, thereby greatly improving target-to-background ratios (TBR) [1]. One common approach for activating optical probes is to utilize endogenous enzymatic activity found in the tumor microenvironment which is either not present in normal tissue or found in much lower concentrations. Such enzyme-activatable optical probes are amenable to application by topical spraying during surgical and endoscopic procedures and can be used to identify lesions and their margins for resection [2].

γ-glutamyl hydroxymethyl rhodamine green (gGlu-HMRG) is an activatable optical probe that produces the green fluorescent product, HMRG, after exposure to γ-glutamyltranspeptidase (GGT), a cell surface-associated (or bound) enzyme involved in cellular glutathione homeostasis. GGT is overexpressed in several human tumors, including cervical and ovarian cancers [3–7]. gGlu-HMRG has been reported to detect peritoneal ovarian cancer metastases (POCM) within 10 min of topical application in animal models because of its rapid and strong activation upon contact with GGT. However, while gGlu-HMRG was successful in models using some ovarian cancer cell lines such as SHIN3, it failed to visualize metastases in other cell lines such as SKOV3 and OVCAR3 because of their lower GGT activity. In addition, even when fluorescence was activated, it tended to be short-lived in SHIN3 cells, suggesting that detectability of cancer may decrease with time using gGlu-HMRG [3].

**Scheme 1:**
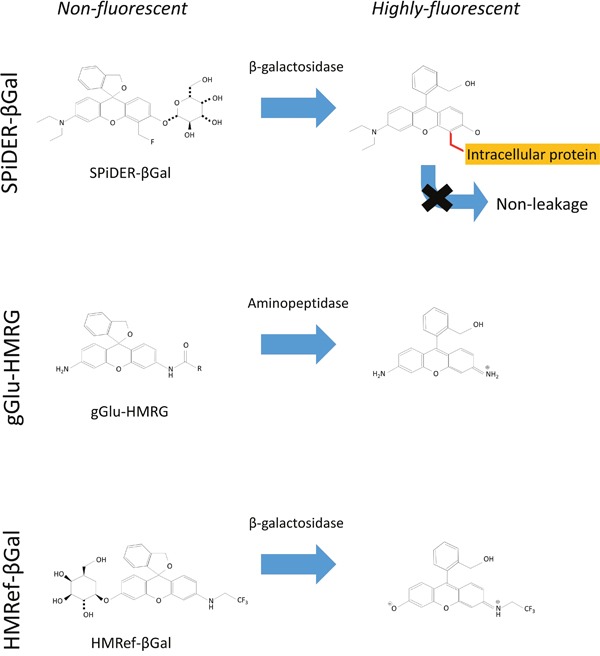
Chemical structure of each probe

β-galactosidase catalyzes the hydrolysis of lactose into glucose and galactose, and its enhanced enzymatic activity in primary ovarian cancers compared with normal ovaries has been reported [8, 9]. X-gal staining has been the most popular technique to determine whether cells express β-galactosidase or not. The X-gal technique is indeed widely used, but its substrate generally shows relatively poor cell permeability [10]. Further, fluorescein di-O-β-galactoside is well-known to be membrane impermeable and cannot be used to take fluorescence images of living cells without a severe loading technique, such as hypotonic shock [11, 12]. Some activatable probes which allow the real time imaging of β-galactosidase activity in living cells because of membrane permeability due to its lower hydrophilicity have been reported [9, 12, 13]. HMRef-βGal, which is the original probe activated by β-galactosidase, behaves with similar kinetics to gGlu-HMRG probe but produced weaker fluorescence than gGlu-HMRG [9] (Scheme 1, Supplementary Figure 1). In addition, the first generation of β-galactosidase activated probe tends to leak out of cells during prolonged incubation [13]. SPiDER-βGal is a newly developed fluorogenic β-galactosidase substrate suitable for labeling live cells in culture, as well as in living tissues. In contrast to the first generation agent, this fluorescent probe exhibited dramatic activation of fluorescence upon reaction with β-galactosidase, which persisted as the probe internalized and was anchored to intracellular proteins, enabling single cell resolution [14]. Its capacity to internalize, bind and be retained intracellularly likely accounts for the persistence of fluorescence over time.

Herein, we compare the fluorescence signal and its kinetics generated by SPiDER-βGal activated by β-galactosidase to gGlu-HMRG activated by GGT, using ovarian cancer cell lines both *in vitro* and *ex vivo* tissue imaging.

## RESULTS

### *In vitro* fluorescence imaging

**Figure 1:**
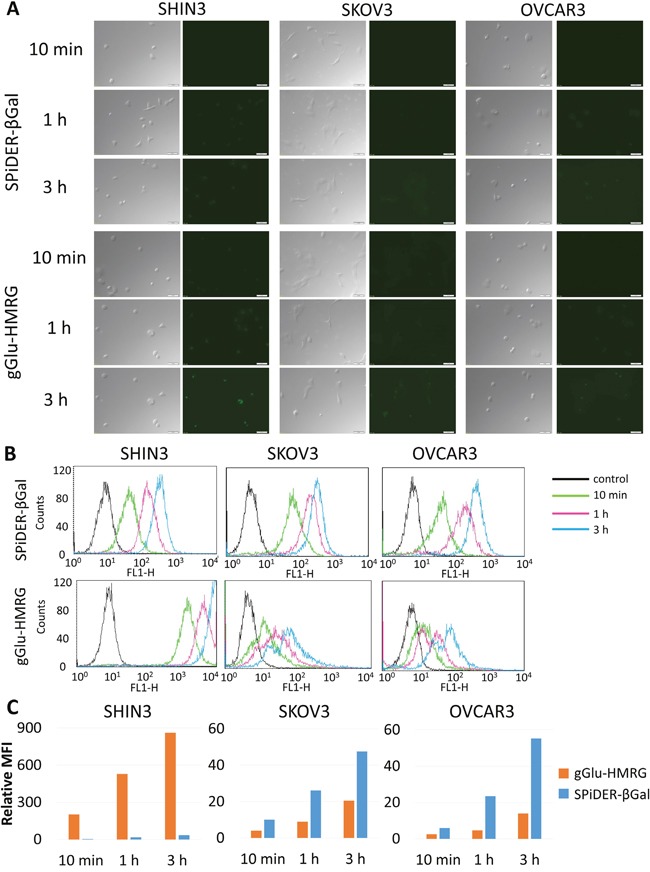
**(A)** Fluorescence microscopy studies. SHIN3, SKOV3, and OVCAR3 cells were incubated with SPiDER-βGal and gGlu-HMRG for 10 min, 1, and 3 h. After 3 h incubation of gGlu-HMRG, SHIN3 cells showed stronger fluorescence compared to those incubated with SPiDER-βGal. SKOV3 and OVCAR3 cells incubated with SPiDER-βGal seemed to show comparable fluorescence compared to those incubated with gGlu-HMRG. **(B)** Flow cytometric analysis. One representative individual is shown. **(C)** Relative MFI of gGlu-HMRG in SHIN3 cells was significantly higher compared to that of SPiDER-βGal regardless of incubation time while relative MFI of SPiDER-βGal in SKOV3 and OVCAR3 cells was significantly higher compared to that of gGlu-HMRG regardless of incubation time.

SHIN3 cells showed stronger activation and accumulation of gGlu-HMRG compared to SPiDER-βGal regardless of incubation time as confirmed by 2D and confocal fluorescence microscopy and flow cytometry (Figure 1A, 1B, Supplementary 1 and 3 videos). The relative MFI of gGlu-HMRG was also significantly higher compared to that of SPiDER-βGal, regardless of incubation time (*p* < 0.01 for all incubation times) (Figure 1C).

SKOV3 and OVCAR3 cells showed almost comparable activation and accumulation of gGlu-HMRG compared to SPiDER-βGal regardless of incubation time although fluorescent signal was quite low compared to that of gGlu-HMRG using SHIN3 cells (Figure 1A, 1B, Supplementary 2 and 4 videos). The relative MFI of gGlu-HMRG was significantly lower than SPiDER-βGal, regardless of incubation time using SKOV3 and OVCAR3 (*p* < 0.01 for all incubation times) (Figure 1C).

### Persistence of fluorescence signal *in vitro*

#### SHIN3 cells

**Figure 2:**
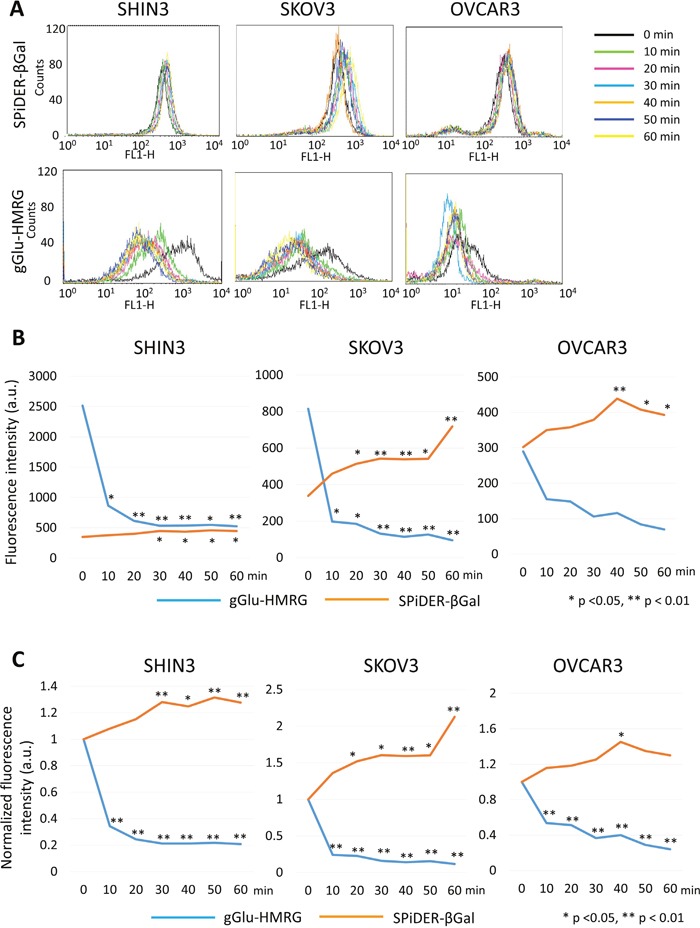
Flow cytometric analysis regarding preservation of fluorescence signal **(A)** One representative individual is shown. **(B)** Time fluorescence intensity curve of each cell line. **(C)** Time normalized fluorescence intensity curve of each cell line.

Removal of gGlu-HMRG resulted in a rapid initial decrease of fluorescence intensity in SHIN3 cells followed by a more gradual decrease up to 30 min before it plateaued (*p* = 0.02 at 10 min, and < 0.01 at 20, 30, 40, 50, and 60 min after removal of gGlu-HMRG, respectively). On the other hand, fluorescence intensity increased gradually up to 60 min after removal of SPiDER-βGal (*p* = 0.54, 0.24, 0.02, 0.04, 0.01, and 0.02 at 10, 20, 30, 40, 50, and 60 min after removal of SPiDER-βGal, respectively) (Figure 2A and 2B).

Similarly, normalized fluorescence intensity decreased rapidly after removal of gGlu-HMRG (*p* < 0.01 at all time points) while normalized fluorescence intensity increased gradually after removal of SPiDER-βGal (*p* = 0.46, 0.14, < 0.01, 0.01, < 0.01, and < 0.01 at 10, 20, 30, 40, 50, and 60 min after removal of SPiDER-βGal, respectively) (Figure 2C).

#### SKOV3 cells

In SKOV3 cells removal of gGlu-HMRG resulted in a rapid decrease of fluorescence intensity followed by a gradual decrease (*p* = 0.01 at 10 and 20 min, and < 0.01 at 30, 40, 50, and 60 min after removal of gGlu-HMRG, respectively). On the other hand, there was a gradual increase in fluorescence intensity after removal of SPiDER-βGal (*p* = 0.11, 0.02, < 0.01, < 0.01, 0.01, and < 0.01 at 10, 20, 30, 40, 50, and 60 min after removal of SPiDER-βGal, respectively) (Figure 2A and 2B).

Similarly, normalized fluorescence intensity decreased rapidly after removal of gGlu-HMRG (*p* < 0.01 at all time points) while normalized fluorescence intensity increased gradually up to 60 min after removal of SPiDER-βGal (*p* = 0.14, 0.03, 0.01, < 0.01, 0.02, and < 0.01 at 10, 20, 30, 40, 50, and 60 min after removal of SPiDER-βGal, respectively) (Figure 2C).

#### OVCAR3 cells

In OVCAR3 cells fluorescence intensity decreased gradually after removal of gGlu-HMRG without reaching significance (*p* = 0.26, 0.27, 0.11, 0.13, 0.07, and 0.05 at 10, 20, 30, 40, 50, and 60 min after removal of gGlu-HMRG, respectively). On the other hand, fluorescence intensity gradually increased after removal of SPiDER-βGal up to 40 min followed by slight decrease (*p* = 0.27, 0.19, 0.06, < 0.01, 0.01, and 0.02 at 10, 20, 30, 40, 50, and 60 min after removal of SPiDER-βGal, respectively) (Figure 2A and 2B).

Normalized fluorescence intensity decreased gradually after removal of gGlu-HMRG (*p* < 0.01 at all time points) while normalized fluorescence intensity increased gradually up to 40 min after removal of SPiDER-βGal (*p* = 0.45, 0.38, 0.23, 0.03, 0.10, and 0.19 at 10, 20, 30, 40, 50, and 60 min after removal of SPiDER-βGal, respectively) (Figure 2C).

### *Ex vivo* activatable imaging of fresh tumors

**Figure 3:**
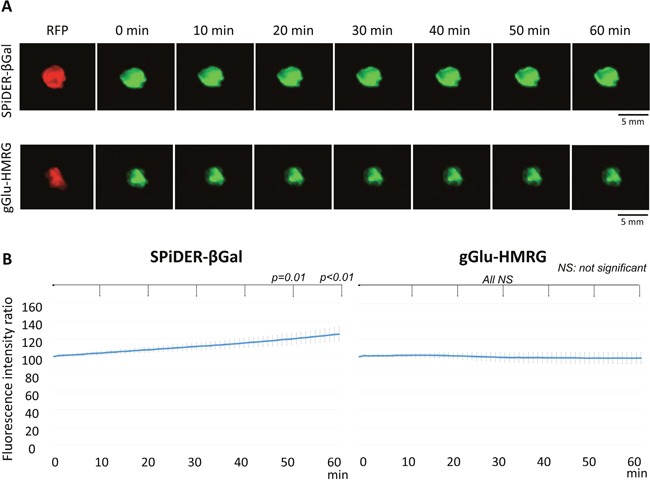
**(A)** Fluorescence images after removal of the probe and RFP image of the extracted tumor. Fluorescence of the tumor with SPiDER-βGal was well preserved while fluorescence of the tumor with gGlu-HMRG decreased resulting in indistinct tumor margins. **(B)** Time fluorescence intensity ratio curve of the extracted tumors after removal of each probe. Data are mean fluorescence intensity ratio ± SEM of tumors at different time points.

The fluorescence intensity ratio of extracted SHIN3-RFP tumors increased gradually after removal of SPiDER-βGal (*p* = 0.65, 0.39, 0.19, 0.06, 0.01, and < 0.01 at 10, 20, 30, 40, 50, and 60 min after removal of SPiDER-βGal, respectively). Additionally, the margin of the fluorescence positive area was clear, with high contrast up to 60 min (Figure 3). On the other hand, the fluorescence intensity ratio of extracted SHIN3-RFP tumors decreased gradually after removal of gGlu-HMRG although it did not reach significance (*p* = 0.79, 0.82, 0.88, 0.90, 0.91, and 0.91 at 10, 20, 30, 40, 50, and 60 min after removal of gGlu-HMRG, respectively). Moreover, the margin became unclear on the later time points (Figure 3).

## DISCUSSION

gGlu-HMRG demonstrates strong and rapid fluorescence in *in vitro* studies using SHIN3 ovarian cancer cells which surpasses that of SPiDER-βGal. In SKOV3 and OVCAR3 ovarian cancer cell lines the two probes showed lower but comparable activation; however, the relative MFI found for SPiDER-βGal using SKOV3 and OVCAR3 cells was significantly higher compared to gGlu-HMRG, regardless of incubation time (10 min, and 1 and 3 h). HMRef-βGal, another β-galactosidase activated probe, has also been reported to visualize metastases as small as < 1 mm in diameter in mouse models of POCM including SKOV3 and OVCAR3 [9]. Thus, it appears that β-galactosidase activated probes including SPiDER-βGal and HMRef-βGal have an advantage for detecting POCM caused by SKOV3 and OVCAR3 cell lines compared to gGlu-HMRG [3].

The persistence of fluorescence after activation is an important practical consideration. If the signal dissipates too rapidly, the probe becomes less useful from a translational perspective. To determine the kinetics of fluorescence signal after removal of the probe we evaluated temporal changes in fluorescence signal *in vitro* and *ex vivo* tissue imaging. gGlu-HMRG demonstrated a rapid decrease of fluorescence intensity after removal of the probe regardless of cell line type *in vitro*. For instance, using SHIN3 cells, the fluorescence intensity decreased > 50% within 10 min of probe removal. *Ex vivo* tissue imaging results also showed a gradual decrease of fluorescence intensity after removal of gGlu-HMRG. For detecting all tiny cancer foci a probe is required to maintain a high TBR over time or otherwise lesions may fade from view. Thus, gGlu-HMRG could result in decreased detection of tumor foci with time.

The SPiDER-βGal probe demonstrated preservation and even increases of signal both in *in vitro* and *ex vivo* tissue imaging. This preservation of fluorescence signal is thought to be due to its internalization within cells followed by its anchoring to intracellular proteins. Moreover, fluorescence signal increased gradually even after removal of SPiDER-βGal. Doura et al. reported that fluorescence derived from SPiDER-βGal increased with time until 120 min, the end of observation, *in vitro* [14], suggesting that it happens slowly in a few hours to form the protein anchored molecule from SPiDER-βGal. Thus, we speculated that SPiDER-βGal which remained inside of cell was activated by β-galactosidase and bound to intracellular protein gradually, leading to a progressive increase in fluorescence intensity even after removal of SPiDER-βGal. Taken together, we conclude that SPiDER-βGal is a promising probe that has advantages over gGlu-HMRG, especially with regard to maintaining TBR over time. Since different molecular probes which are activated by β-galactosidase, yet made based on different fluorophore emitting different colors of light such as cyanine were reported [15], we will compare the probes with SPiDER-βGal in future study.

Complete resection of tumor is essential for curative treatment of cancer although evaluation of tumor margin is difficult to be determined by naked eyes. Intraoperative frozen section analysis (IFSA) is widely used clinically for accurate evaluation of tumor margin although it takes 20 to 30 min. However, IFSA has some problems such as manpower, cost and time to conduct total-circumferential examination. In addition, many surgeons and pathologists select only a few samples from the margins for IFSA, resulting in false negatives [16, 17]. Therefore, optical fluorescence-guided imaging has the potential to aid not only in tumor detection but also in determining the status of surgical margins in real time for improving outcomes because fluorescence derived from optical probes can evaluate tumor margin as a whole on site at the surgical suite within 5 to 10 min [3, 16]. From our *ex vivo* tissue imaging results the decrease in fluorescence seen with gGlu-HMRG led to decreases in the conspicuity of the margin of the tumor on later time points. This made defining the tumor margin more difficult even risking a false negative diagnosis. On the other hand, fluorescence of the tumor with SPiDER-βGal gradually increased over time, suggesting that SPiDER-βGal may be better at defining tumor margins. Extracted tumors showed sufficient fluorescence 30 min after spraying SPiDER-βGal, indicating that surgeons must wait about 30 min to see optimal results after spraying SPiDER-βGal. However, this waiting time is not long compared to the time needed for IFSA. Thus, we believe accurate evaluation of tumor margin using SPiDER-βGal should contribute to curative treatment of cancer clinically.

Genetic reporters have been reported to illuminate cancer selectively *in vivo* [18–25]. However, ethical consideration is needed clinically because genetic reporters needs virus administration. In addition, to clarify the preclinical effect, orthotopic mouse models of ovarian cancer, peritoneal ovarian cancer metastases, offer important advantages over the subcutaneous models [26–28]. Yet, tumors of peritoneal ovarian cancer metastases in mice are too tiny for evaluation of tumor margin. Thus, we chose the subcutaneous models.

In conclusion, we describe a new activatable probe, SPiDER-βGal, activated by β-galactosidase that remains inside cells, anchoring itself to intracellular proteins. SPiDER-βGal visualized ovarian cancer cells regardless of cell line type. The advantages of this probe are its increasing TBR with time and improved contrast at the tumor margin. Thus, we suggest that SPiDER-βGal has potential as a new alternative to existing activatable probes for optical fluorescence-guided imaging.

## MATERIALS AND METHODS

### Reagents

SPiDER-βGal was obtained from Dojindo Molecular Technologies, Inc. (Rockville, MD, USA) [14]. gGlu-HMRG, a GGT activated fluorescence probe was synthesized as described previously [3].

### Cell lines and culture

The established ovarian cancer cell lines, SHIN3, SKOV3, and OVCAR3 were used for *in vitro* fluorescence microscopy and flow cytometry. High expression of GGT has been reported in SHIN3 while SKOV3 and OVCAR3 showed lower GGT activity [3]. SHIN3 and OVCAR3 showed high expression of β-galactosidase while SKOV3 showed moderate expression of β-galactosidase [9]. SHIN3-DsRed, in which the red fluorescent protein (RFP DsRed2)-expressing plasmid (Clontech Laboratories, Mountain View, CA, USA) was previously transfected, was used for *ex vivo* tumor imaging [29]. Cell lines were grown in RPMI 1640 supplemented with 10 % FBS and 1 % penicillin-streptomycin (Life Technologies) in tissue culture flasks in a humidified incubator at 37°C in an atmosphere of 95 % air and 5 % carbon dioxide.

### *In vitro* fluorescence microscopy and flow cytometry

To compare fluorescence intensities of SPiDER-βGal or gGlu-HMRG, we performed fluorescence microscopy. 4 × 10^4^ cells from each cell line were plated on a culture well covered by a glass cover slip and incubated in culture media for 24 h. SPiDER-βGal or gGlu-HMRG (1 *μ*M) was added to the culture medium and incubated for 10 min, 1, and 3 h. After incubation, cells were washed once with phosphate-buffered saline solution (PBS), and fluorescence microscopy was performed using an Olympus BX61 microscope (Olympus America, Inc., Melville, NY) equipped with the following filters: excitation wavelength range 450–490 nm and emission wavelength range 500–550 nm. Transmitted light differential interference contrast (DIC) images were obtained at the same time.

For evaluating localization of fluorescence signal in cells in detail, 4 × 10^4^ SHIN3 or OVCAR3 cells were seeded on cover-glass-bottomed dishes and incubated in culture media for 24 h. SPiDER-βGal or gGlu-HMRG (1 *μ*M) was added to the culture medium and incubated for 30 min. After incubation, cells were washed once with PBS, and confocal fluorescence microscopy at x600 magnification was performed using an Olympus IX81 disk-scanning confocal microscope (Olympus America, Inc., Melville, NY) equipped with the following filters: excitation wavelength range 470–495 nm and emission wavelength range 510–550 nm, and step size: 0.6 *μ*m.

For flow cytometry, 1 × 10^5^ cells from each cell line were plated in a 24-chamber culture well and incubated for 24 h. SPiDER-βGal or gGlu-HMRG (1 *μ*M) was added to the culture medium, and cells were incubated for 10 min, 1, and 3 h. A 488-nm argon ion laser was used for excitation. Signals from cells were collected with a 515 to 545 nm band-pass filter. Cells were analyzed using a FACS Calibur (BD BioSciences, San Jose, CA, USA). Relative mean fluorescence intensity (MFI) was quantified as the ratio MFI_target_ to MFI_control_ using CellQuest software (BD BioScience). Samples were assayed three times in duplicate.

### Kinetics of fluorescence signal *in vitro*

To determine whether fluorescence signal is preserved after removal of the probe, we evaluated the temporal change in fluorescence signal *in vitro* using flow cytometry. 1 × 10^5^ cells from each cell line were plated in a 24-chamber culture well and incubated for 24 h. SPiDER-βGal or gGlu-HMRG (1 *μ*M) was added to the culture medium, and cells were incubated for 3 h. After washing with PBS twice, medium was replaced with fresh culture medium without the probe and incubated for 0, 10, 20, 30, 40, 50, and 60 min at 37°C. Signals from cells were collected and analyzed in the same manner as that described above using a FACS Calibur. MFI was calculated using CellQuest software. Normalized fluorescence intensity was calculated by dividing each MFI value by the 0 min value obtained without the probe. Samples were assayed three times in duplicate.

### Animal model

All procedures were performed in compliance with the Guide for the Care and Use of Laboratory Animals [30] and approved by the local Animal Care and Use Committee. Six- to 8-week old female homozygote athymic nude mice were purchased from Charles River (National Cancer Institute, Frederick, MD).

A subcutaneous injection of 2 × 10^6^ SHIN3-DsRed cells suspended in 200 *μ*l of PBS was performed in the right and left dorsi of mice. The mice were evaluated 7–10 days after injection of the cells.

### *Ex vivo* activatable imaging of fresh tumors

Mice with tumors were euthanized by carbon dioxide inhalation. Immediately after euthanasia, subcutaneous tumors were extracted. Dilute aqueous solutions of SPiDER-βGal or gGlu-HMRG (20 *μ*l of 100 *μ*M) were sprayed on the extracted tumor (*n* ≧ 4 for each group). Tumors were heated to 37°C via heating pad for 30 min after spray application of the probe because activity of β-galactosidase is temperature dependent [31–33]. To observe the kinetics of fluorescence signal retention within tumors, tumors were rinsed with PBS twice after heating, and then excess PBS was removed.

For evaluation of red fluorescence indicating the presence of tumor, images were acquired using the Maestro *In-Vivo* Imaging System (Cri, Woburn, MA, USA). The following filter set was used: a band-path filter from 503 to 555 nm for excitation light and a long-pass filter over 645 nm for emission light. The tunable emission filter was automatically stepped in 10 nm increments from 600 to 800 nm at constant exposure times. The spectral fluorescence images consisting of spectra from autofluorescence and RFP were then unmixed, based on their known spectral patterns using commercial software (Maestro software; CRi).

Serial fluorescence imaging of the tumor was performed after rinsing with PBS. A portable fluorescence camera (Discovery INDEC BioSystems, Santa Clara, CA, USA) was utilized [34] with the following filter set: band-pass filter from 450 to 490 nm for excitation light and from 511 to 551 nm for emission light, with an exposure time of 50 msec. Extracted specimens were placed on a non-fluorescent plate. Real-time fluorescence images were recorded every 1 min between 0 and 60 min at room temperature. Regions of interest (ROIs) were drawn within the tumor nodules depicted by the RFP images and then the average fluorescence intensity of each ROI was measured. Fluorescence intensity ratio was calculated from the average fluorescence intensity at each time point divided by that at baseline. All fluorescence images were analyzed with ImageJ software (http://rsb.info.nih.gov/ij/).

### Statistical analyses

Statistical analyses were performed with JMP 10 software (SAS Institute, Cary, NC). The difference of relative MFI between SPiDER-βGal and gGlu-HMRG was determined with the two-sided Mann–Whitney's *U* test. The differences in fluorescence intensity every 10 min compared to the value at 0 min were compared using Dunnett's multiple comparison. Differences of *p* < 0.05 were considered statistically significant.

## SUPPLEMENTARY FIGURE AND VIDEOS











## References

[1] Kobayashi H, Choyke PL Target-cancer-cell-specific activatable fluorescence imaging probes: rational design and in vivo applications. Acc Chem Res.

[2] Razgulin A, Ma N, Rao J Strategies for in vivo imaging of enzyme activity: an overview and recent advances. Chem Soc Rev.

[3] Urano Y, Sakabe M, Kosaka N, Ogawa M, Mitsunaga M, Asanuma D, Kamiya M, Young MR, Nagano T, Choyke PL, Kobayashi H Rapid cancer detection by topically spraying a gamma-glutamyltranspeptidase-activated fluorescent probe. Sci Transl Med.

[4] Hanigan MH, Frierson HF, Brown JE, Lovell MA, Taylor PT Human ovarian tumors express gamma-glutamyl transpeptidase. Cancer Res.

[5] Yao D, Jiang D, Huang Z, Lu J, Tao Q, Yu Z, Meng X Abnormal expression of hepatoma specific gamma-glutamyl transferase and alteration of gamma-glutamyl transferase gene methylation status in patients with hepatocellular carcinoma. Cancer.

[6] Schafer C, Fels C, Brucke M, Holzhausen HJ, Bahn H, Wellman M, Visvikis A, Fischer P, Rainov NG Gamma-glutamyl transferase expression in higher-grade astrocytic glioma. Acta Oncol.

[7] Hanigan MH, Frierson HF, Swanson PE, De Young BR Altered expression of gamma-glutamyl transpeptidase in human tumors. Hum Pathol.

[8] Chatterjee SK, Bhattacharya M, Barlow JJ Glycosyltransferase and glycosidase activities in ovarian cancer patients. Cancer Res.

[9] Asanuma D, Sakabe M, Kamiya M, Yamamoto K, Hiratake J, Ogawa M, Kosaka N, Choyke PL, Nagano T, Kobayashi H, Urano Y Sensitive beta-galactosidase-targeting fluorescence probe for visualizing small peritoneal metastatic tumours in vivo. Nat Commun.

[10] Nirenberg S, Cepko C Targeted ablation of diverse cell classes in the nervous system in vivo. J Neurosci.

[11] Nolan GP, Fiering S, Nicolas JF, Herzenberg LA Fluorescence-activated cell analysis and sorting of viable mammalian cells based on beta-D-galactosidase activity after transduction of Escherichia coli lacZ. Proc Natl Acad Sci U S A.

[12] Urano Y, Kamiya M, Kanda K, Ueno T, Hirose K, Nagano T Evolution of fluorescein as a platform for finely tunable fluorescence probes. J Am Chem Soc.

[13] Kamiya M, Asanuma D, Kuranaga E, Takeishi A, Sakabe M, Miura M, Nagano T, Urano Y β-Galactosidase fluorescence probe with improved cellular accumulation based on a spirocyclized rhodol scaffold. J Am Chem Soc.

[14] Doura T, Kamiya M, Obata F, Yamaguchi Y, Hiyama TY, Matsuda T, Fukamizu A, Noda M, Miura M, Urano Y Detection of LacZ-Positive Cells in Living Tissue with Single-Cell Resolution. Angew Chem Int Ed Engl.

[15] Han J, Han MS, Tung CH A fluorogenic probe for β-galactosidase activity imaging in living cells. Mol Biosyst.

[16] Ueo H, Shinden Y, Tobo T, Gamachi A, Udo M, Komatsu H, Nambara S, Saito T, Ueda M, Hirata H, Sakimura S, Takano Y, Uchi R Rapid intraoperative visualization of breast lesions with gamma-glutamyl hydroxymethyl rhodamine green. Sci Rep.

[17] Fukamachi K, Ishida T, Usami S, Takeda M, Watanabe M, Sasano H, Ohuchi N Total-circumference intraoperative frozen section analysis reduces margin-positive rate in breast-conservation surgery. Jpn J Clin Oncol.

[18] Kishimoto H, Aki R, Urata Y, Bouvet M, Momiyama M, Tanaka N, Fujiwara T, Hoffman RM Tumor-selective, adenoviral-mediated GFP genetic labeling of human cancer in the live mouse reports future recurrence after resection. Cell Cycle.

[19] Yano S, Miwa S, Kishimoto H, Uehara F, Tazawa H, Toneri M, Hiroshima Y, Yamamoto M, Urata Y, Kagawa S, Bouvet M, Fujiwara T, Hoffman RM Targeting tumors with a killer-reporter adenovirus for curative fluorescence-guided surgery of soft-tissue sarcoma. Oncotarget.

[20] Yano S, Miwa S, Kishimoto H, Toneri M, Hiroshima Y, Yamamoto M, Bouvet M, Urata Y, Tazawa H, Kagawa S, Fujiwara T, Hoffman RM Experimental Curative Fluorescence-guided Surgery of Highly Invasive Glioblastoma Multiforme Selectively Labeled With a Killer-reporter Adenovirus. Mol Ther.

[21] Yano S, Zhang Y, Miwa S, Kishimoto H, Urata Y, Bouvet M, Kagawa S, Fujiwara T, Hoffman RM Precise navigation surgery of tumours in the lung in mouse models enabled by in situ fluorescence labelling with a killer-reporter adenovirus. BMJ Open Respir Res.

[22] Yano S, Takehara K, Miwa S, Kishimoto H, Hiroshima Y, Murakami T, Urata Y, Kagawa S, Bouvet M, Fujiwara T, Hoffman RM Improved Resection and Outcome of Colon-Cancer Liver Metastasis with Fluorescence-Guided Surgery Using In Situ GFP Labeling with a Telomerase-Dependent Adenovirus in an Orthotopic Mouse Model. PLoS One.

[23] Yano S, Miwa S, Kishimoto H, Urata Y, Tazawa H, Kagawa S, Bouvet M, Fujiwara T, Hoffman RM Eradication of osteosarcoma by fluorescence-guided surgery with tumor labeling by a killer-reporter adenovirus. J Orthop Res.

[24] Yano S, Takehara K, Miwa S, Kishimoto H, Tazawa H, Urata Y, Kagawa S, Bouvet M, Fujiwara T, Hoffman RM Fluorescence-guided surgery of a highly-metastatic variant of human triple-negative breast cancer targeted with a cancer-specific GFP adenovirus prevents recurrence. Oncotarget.

[25] Yano S, Hiroshima Y, Maawy A, Kishimoto H, Suetsugu A, Miwa S, Toneri M, Yamamoto M, Katz MH, Fleming JB, Urata Y, Tazawa H, Kagawa S Color-coding cancer and stromal cells with genetic reporters in a patient-derived orthotopic xenograft (PDOX) model of pancreatic cancer enhances fluorescence-guided surgery. Cancer Gene Ther.

[26] Matsumoto Y, Miwa S, Zhang Y, Hiroshima Y, Yano S, Uehara F, Yamamoto M, Toneri M, Bouvet M, Matsubara H, Hoffman RM, Zhao M Efficacy of tumor-targeting Salmonella typhimurium A1-R on nude mouse models of metastatic and disseminated human ovarian cancer. J Cell Biochem.

[27] Matsumoto Y, Miwa S, Zhang Y, Zhao M, Yano S, Uehara F, Yamamoto M, Hiroshima Y, Toneri M, Bouvet M, Matsubara H, Tsuchiya H, Hoffman RM Intraperitoneal administration of tumor-targeting Salmonella typhimurium A1-R inhibits disseminated human ovarian cancer and extends survival in nude mice. Oncotarget.

[28] Hoffman RM Patient-derived orthotopic xenografts: better mimic of metastasis than subcutaneous xenografts. Nat Rev Cancer.

[29] Hama Y, Urano Y, Koyama Y, Choyke PL, Kobayashi H D-galactose receptor-targeted in vivo spectral fluorescence imaging of peritoneal metastasis using galactosamin-conjugated serum albumin-rhodamine green. J Biomed Opt.

[30] Guide for the Care and Use of Laboratory Animals (1996).

[31] Wang GX, Gao Y, Hu B, Lu XL, Liu XY, Jiao BH A novel cold-adapted beta-galactosidase isolated from Halomonas sp. S62: gene cloning, purification and enzymatic characterization. World J Microbiol Biotechnol.

[32] Cieslinski H, Kur J, Bialkowska A, Baran I, Makowski K, Turkiewicz M Cloning, expression, and purification of a recombinant cold-adapted beta-galactosidase from antarctic bacterium Pseudoalteromonas sp. 22b. Protein Expr Purif.

[33] Trimbur DE, Gutshall KR, Prema P, Brenchley JE Characterization of a psychrotrophic Arthrobacter gene and its cold-active beta-galactosidase. Appl Environ Microbiol.

[34] Kakareka JW, McCann TE, Kosaka N, Mitsunaga M, Morgan NY, Pohida TJ, Choyke PL, Kobayashi H A portable fluorescence camera for testing surgical specimens in the operating room: description and early evaluation. Mol Imaging Biol.

