# Supplementary material for: A topically-sprayable, activatable fluorescent and retaining probe, SPiDER-βGal for detecting cancer: Advantages of anchoring to cellular proteins after activation
Source: Oncotarget. 2017 Apr 13;8(24):39512–21. doi: 10.18632/oncotarget.17080 (PMC5503628; doi:10.18632/oncotarget.17080)
Supplement: Supplementary file 1 [file oncotarget-08-39512-s001.pdf]

A topically-sprayable, activatable fluorescent and retaining probe, SPiDER-βGal for detecting cancer: Advantages of anchoring to cellular proteins after activation

SUPPLEMENTARY DATA

RESULTS

All cell lines showed stronger activation and accumulation of gGlu-HMRG compared to HMRef-βGal regardless of incubation time as confirmed by fluorescence microscopy and flow cytometry (Supplementary Figure 1A and 1B). The relative MFI of gGlu-HMRG was also significantly higher compared to that of HMRef-βGal, regardless of incubation time ($p < 0.01$ for all incubation times of all cell lines) (Supplementary Figure 1C).

MATERIALS AND METHODS

Reagents

gGlu-HMRG, a GGT activated fluorescence probe and HMRef-βGal, a β-galactosidase activated fluorescence probe was synthesized as described previously [3, 9].

Cell lines and culture

The established ovarian cancer cell lines, SHIN3, SKOV3, and OVCAR3 were used for *in vitro* fluorescence microscopy and flow cytometry. Cell lines were grown in RPMI 1640 supplemented with 10 % FBS and 1 % penicillin-streptomycin (Life Technologies) in tissue culture flasks in a humidified incubator at 37° in an atmosphere of 95 % air and 5 % carbon dioxide.

In vitro fluorescence microscopy and flow cytometry

To compare fluorescence intensities of gGlu-HMRG or HMRef-βGal, we performed fluorescence microscopy. 4×10^4 cells from each cell line were plated on a culture well covered by a glass cover slip and incubated in culture media for 24 h. gGlu-HMRG or HMRef-βGal (1 μM) was added to the culture medium and incubated for 10 min, 1, and 3 h. After incubation, cells were washed once with phosphate-buffered saline solution (PBS), and fluorescence microscopy was performed using an Olympus BX61 microscope (Olympus America, Inc., Melville, NY) equipped with the following filters: excitation wavelength range 450–490 nm and emission wavelength range 500–550 nm. Transmitted light differential interference contrast (DIC) images were obtained at the same time.

For flow cytometry, 1×10^5 cells from each cell line were plated in a 24-chamber culture well and incubated for 24 h. gGlu-HMRG or HMRef-βGal (1 μM) was added to the culture medium, and cells were incubated for 10 min, 1, and 3 h. A 488-nm argon ion laser was used for excitation. Signals from cells were collected with a 515 to 545 nm band-pass filter. Cells were analyzed using a FACS Calibur (BD BioSciences, San Jose, CA, USA). Relative mean fluorescence intensity (MFI) was quantified as the ratio MFI_{target} to $MFI_{control}$ using CellQuest software (BD BioScience). Samples were assayed three times in duplicate.

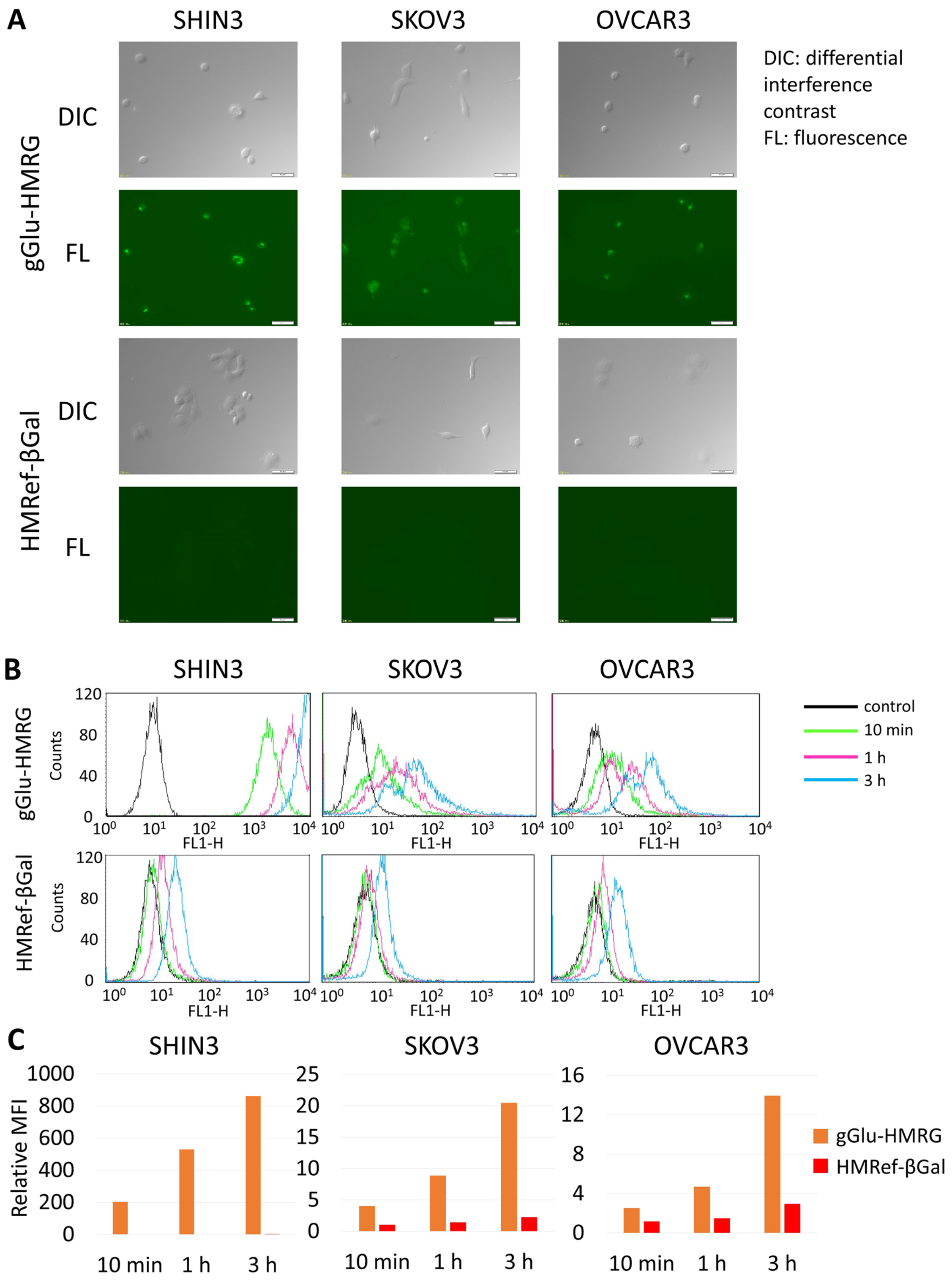

Supplementary Figure 1: (A) Fluorescence microscopy studies. SHIN3, SKOV3, and OVCAR3 cells were incubated with gGlu-HMRG and HMRef-βGal for 3 h. With incubation of gGlu-HMRG all cell lines showed stronger fluorescence compared to those incubated with HMRef-βGal. **(B)** Flow cytometric analysis. One representative individual is shown. **(C)** Relative MFI of gGlu-HMRG in all cell lines was significantly higher compared to that of HMRef-βGal regardless of incubation time.

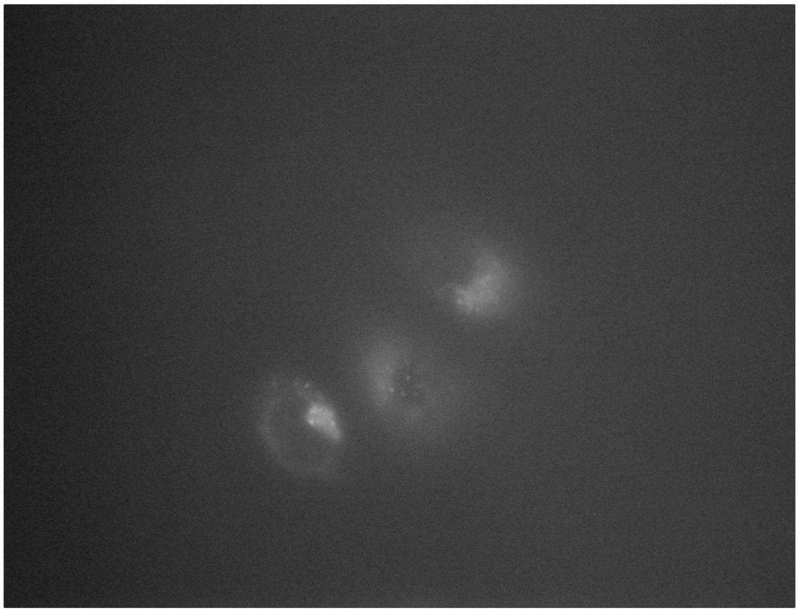

Supplementary Video 1: Confocal fluorescence microscopy study acquired at x600 magnification of SHIN3 cells incubated with SPiDER-βGal for 30 min. Spotty fluorescence signal is clearly shown in the cytoplasm.

See Supplementary Video 1

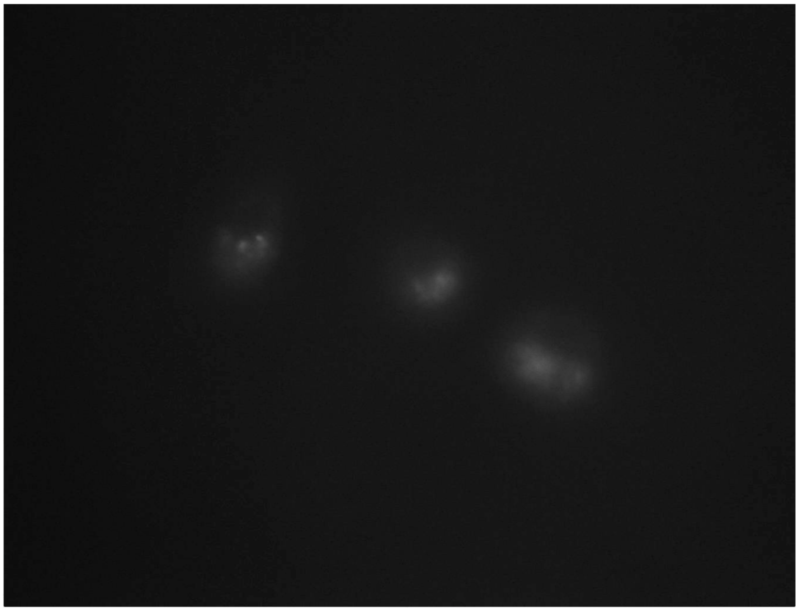

Supplementary Video 2: Confocal fluorescence microscopy study acquired at x600 magnification of OVCAR3 cells incubated with SPiDER-βGal for 30 min. Spotty fluorescence signal is clearly shown in the cytoplasm.

See Supplementary Video 2

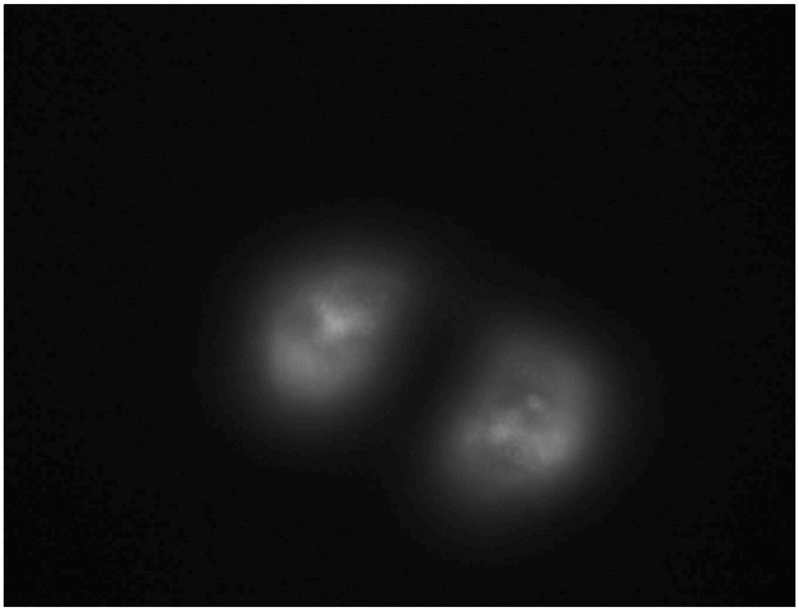

Supplementary Video 3: Confocal fluorescence microscopy study acquired at x600 magnification of SHIN3 cells incubated with gGlu-HMRG for 30 min. Spotty fluorescence signal is clearly shown in the cytoplasm.

See Supplementary Video 3

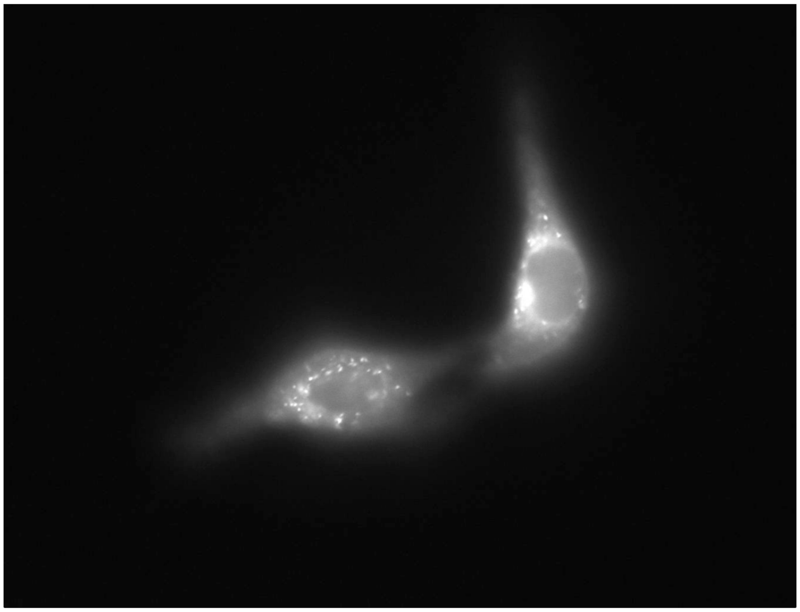

Supplementary Video 4: Confocal fluorescence microscopy study acquired at x600 magnification of OVCAR3 cells incubated with gGlu-HMRG for 30 min. Spotty fluorescence signal is clearly shown in the cytoplasm.

See Supplementary Video 4
